# Recurrent Exacerbations of Chronic Obstructive Pulmonary Disease Reveal Swyer-James-MacLeod Syndrome in a 63-year-old Patient

**DOI:** 10.7759/cureus.12601

**Published:** 2021-01-10

**Authors:** Serafeim Chlapoutakis, Anna Garmpi, Nikolaos Trakas, Christos Damaskos, Vasiliki E Georgakopoulou

**Affiliations:** 1 Department or Thoracic Surgery, Agios Savvas Hospital, Athens, GRC; 2 First Department of Propedeutic Internal Medicine, Laiko General Hospital, Athens Medical School, National and Kapodistrian University of Athens, Athens, GRC; 3 Biochemistry Department, Sismanogleio Hospital, Athens, GRC; 4 Surgery, Laiko General Hospital, Laboratory of Experimental Surgery and Surgical Research “N.S. Christeas”, Athens Medical School, National and Kapodistrian University of Athens, Athens, GRC; 5 Pulmonology Department, Laiko General Hospital, Athens, GRC; 6 First Pulmonology Department, Sismanogleio Hospital, Athens, GRC

**Keywords:** copd, macleod syndrome, swyer james syndrome, dyspnea

## Abstract

Swyer-James-Macleod syndrome is an infrequent clinical condition characterized by unilateral hyperlucent lung as a complication following infectious bronchiolitis obliterans, typically diagnosed during childhood. However, in some patients, the diagnosis may be confirmed in adulthood. The syndrome can be misdiagnosed with other lung disorders such as asthma, pulmonary embolism, and pneumothorax, leading to inappropriate management and worse outcome. We present a case of Swyer-James-MacLeod syndrome, diagnosed in a 63-year-old man, with frequent hospitalisations due to chronic obstructive pulmonary disease (COPD) exacerbations without a history of significant lung infection in childhood. Complications of Swyer-James-MacLeod syndrome include recurrent infections, lung abscess, pneumothorax, and pulmonary hypertension. The syndrome should always be considered in adults with recurrent respiratory infections or pulmonary hyperlucency on chest imaging to prevent a delay in correct diagnosis and improper treatment.

## Introduction

Swyer-James-MacLeod syndrome, also known as unilateral hyperlucent lung syndrome, is a rare clinical entity related to postinfectious childhood bronchiolitis obliterans. Characteristic feature is hypoplasia and/or agenesis of the pulmonary arteries leading to lung parenchyma hypoperfusion [1]. Traditionally, the diagnosis is made in childhood after an investigation for recurrent respiratory infections, but in some cases, patients with little or no bronchiectasis have few symptoms or are asymptomatic and may be undiagnosed until adulthood [2].

Diagnosis in adulthood is relatively less frequent and patients present with dyspnea, decreased exercise tolerance, productive cough, hemoptysis, and recurrent lung infections or it is an accidental finding [3]. The syndrome can mimic other pulmonary diseases such as asthma, pulmonary embolism, and pneumothorax resulting in inappropriate therapy [2,4].

Herein we present a case of Swyer-James-MacLeod syndrome, diagnosed in a 63-year-old man, with frequent hospitalisations due to chronic obstructive pulmonary disease (COPD) exacerbations.

## Case presentation

A 63-year-old male, a current heavy smoker with a smoking history of over 100-pack years, was referred due to complaints of dyspnea at rest and productive cough over the last three days. He had a history of COPD; he was diagnosed the previous year and treated with a combination of inhaled beclomethasone, formoterol, and glycopyrronium, with more of three hospitalizations due to infectious exacerbations the last six months. He had also a history of coronary artery disease with coronary artery bypass done nine years ago and circumflex artery angioplasty four years ago, as well as aortic valve replacement with metallic prosthesis, arterial hypertension, and dyslipidemia. The patient mentioned no severe lung infection during his childhood.

**Figure 1:**
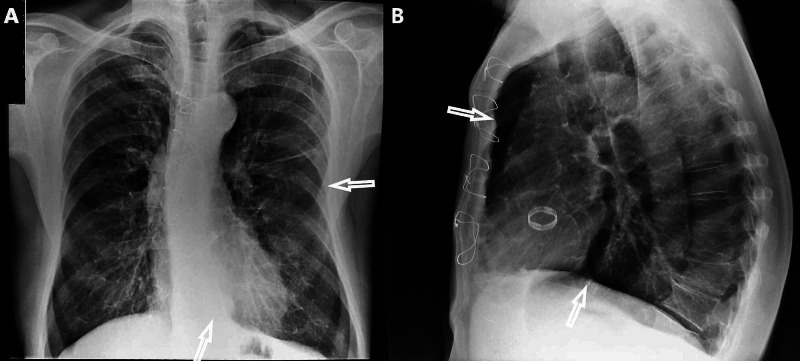
Chest X-ray showing emphysematous changes A. Arrows show horizontal ribs and small size of the heart. B. Arrows show increased retrosternal space and flattened hemidiaphragms.

Clinical examination revealed decreased breath sounds in both lungs and wheezing. Blood pressure was 110/60 mmHg, heart rate was 90 beats per minute, oxygen saturation was 94% on room air, and body temperature 36.5°C. Arterial blood gas analysis revealed partial pressure of oxygen (pO2) 66 mmHg, partial pressure of carbon dioxide (pCO2) 41 mmHg, pH 7.47, and bicarbonate (HCO3−) 29.8 mmol/L on room air. Electrocardiography showed no abnormal findings. Laboratory findings were normal. Chest X-ray showed emphysematous lesions with lungs of large volume, flattened hemidiaphragms, horizontal ribs, small size of the heart, and increased retrosternal space (Figure 1).

**Figure 2:**
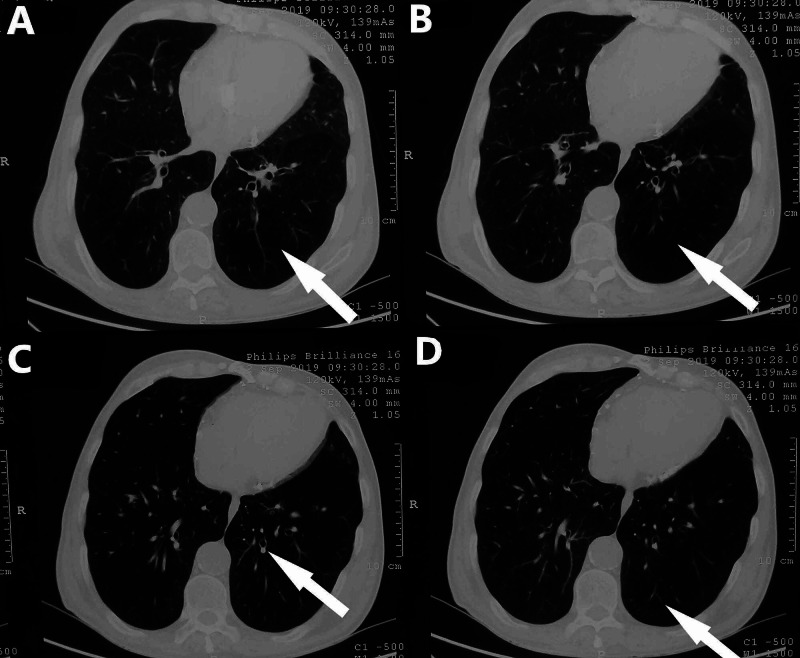
Computed tomography of the chest A, B: Arrows show reduced density in the left lower lobe. C, D: Arrows show bronchiectasis in the left lower lobe.

**Figure 3:**
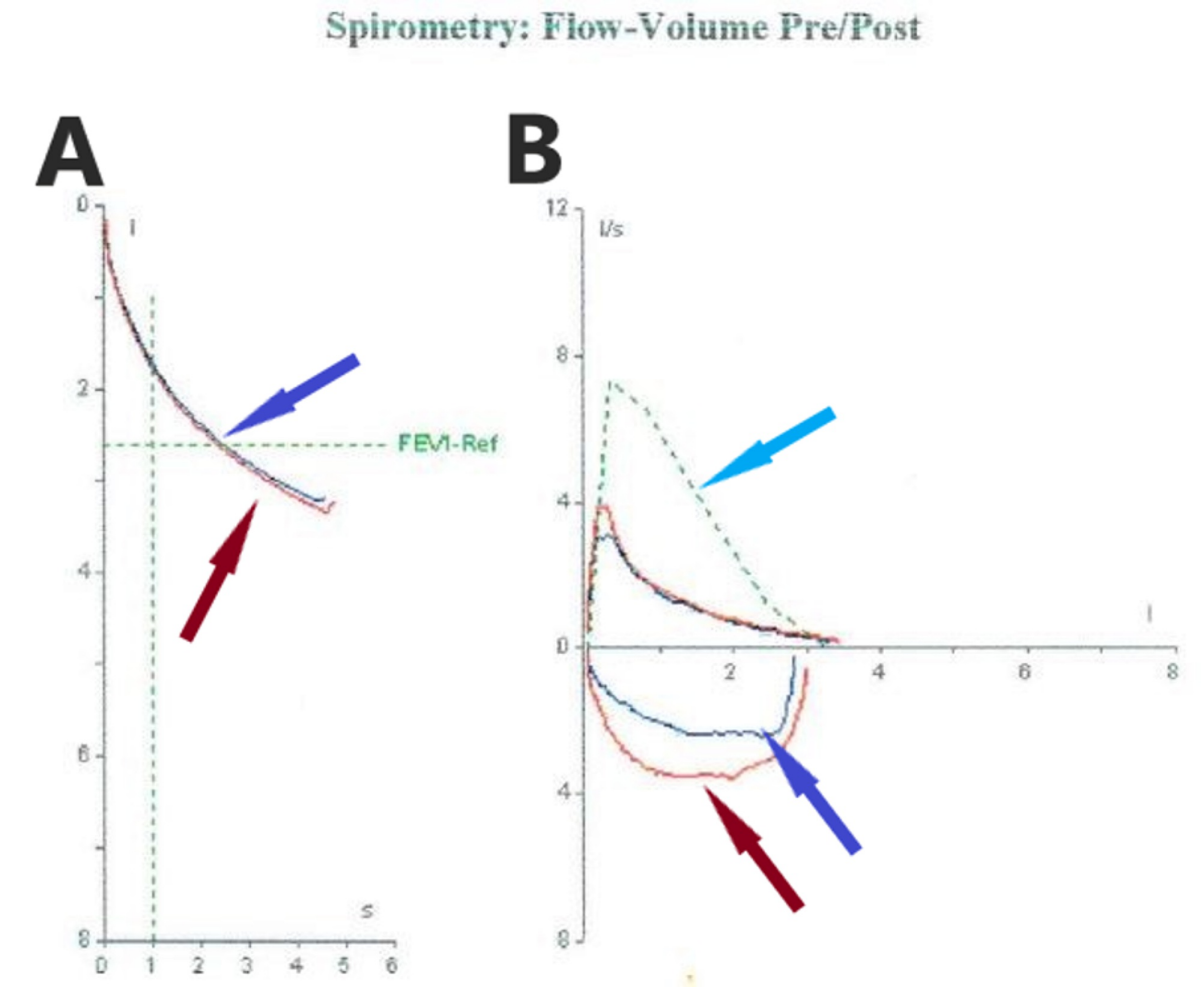
Pulmonary function testing A: Volume/Time Curves (Blue arrow shows pre-bronchodilator curve and red arrow shows post-bronchodilator curve). B: Flow/Volume Curves (Blue arrow shows pre-bronchodilator curve, red arrow shows post-bronchodilator curve and light-blue arrow shows predicted curve).

The patient underwent computed tomography (CT) of the chest in our hospital, which showed bronchiectasis on the left lower lobe. In addition, a reduced density in the same region was observed (Figure 2). Pulmonary function tests (PFTs) revealed a non-reversible mild obstructive pattern. The measured values of forced expiratory volume in one second (FEV1) and forced vital capacity (FVC) were of 65%/67% and 99%/102% of predicted values, pre and post-bronchodilator administration, respectively (Figure 3). Based on clinical manifestations and imaging findings, a diagnosis of Swyer-James-MacLeod syndrome was confirmed.

The patient was treated with nebulized salbutamol/ipratropium, intravenous prednisolone for five days, and empirical antimicrobial therapy with piperacillin-tazobactam while sputum culture did not reveal any microorganism. The patient recovered and was discharged from our hospital with strong advice for smoking cessation and vaccination to prevent pulmonary infections.

## Discussion

Swyer-James-MacLeod syndrome was first reported in 1953 by Swyer and James in a 6-year-old boy [5] and in 1954, Macleod described the syndrome in nine adults with unilateral lung hyperlucency [6]. The prevalence of this syndrome has been reported to be 0.01% [7]. Swyer-James-MacLeod syndrome is typically diagnosed in childhood. Few cases have reported the diagnosis in adults [8]. Swyer-James-MacLeod syndrome is considered to be a postinfective type of bronchiolitis obliterans. Lung infection due to adenovirus, respiratory syncytial virus, influenza A, measles, tuberculosis, Mycoplasma pneumoniae, and Bordetella pertussis has reported to be responsible for the development of the syndrome [9-10]. Interestingly, our patient denied a history of severe lung infection in childhood. It has been reported that some other genetic or environmental components may be responsible for the development of this syndrome [3].

The current case describes a patient with recurrent lung infections and respiratory symptoms, initially attributed only to COPD. The clinical manifestations of Swyer-James-MacLeod syndrome can lead to misdiagnosis of other lung disorders. It has been reported that patients with the syndrome have been misdiagnosed with COPD [3-4, 11], asthma [8], pulmonary embolism [12] and pneumothorax [13]. In addition, they have been improperly treated. Mehra et al. reported a case with the syndrome that has been misdiagnosed with COPD and was treated with numerous courses of steroids for recurrent exacerbations, possibly increasing the risk of infection [11]. Sulaiman et al. reported a case of a 22-year-old female patient with Swyer-James-MacLeod syndrome with an initial diagnosis of pneumothorax who underwent multiple chest tube drainages [13].

Swyer-James-MacLeod syndrome can be diagnosed by the characteristic finding of pulmonary hyperlucency on chest X-ray. Chest CT is the imaging modality of choice in confirming the diagnosis. Chest CT reveals areas of hyperlucency due to reduced pulmonary perfusion of the lung. There may be patchy, low-attenuated, and hypovascular areas among normal parenchyma and bronchiectasis. Ventilation/perfusion scans are a significant diagnostic modality in the diagnosis of the syndrome showing matched ventilation and perfusion defect of the affected lung [8,13]. The current case highlights that a chest X-ray may underestimate the presence of the syndrome and the diagnosis was not confirmed until another complementary imaging study was performed.

Airflow obstruction is generally the typical finding on pulmonary function testing as in our case [14]. Complications of Swyer-James-MacLeod syndrome include recurrent infections, as in our case, especially in regions with bronchiectasis, lung abscess, and pneumothorax [15-17]. Besides, Yuce et al. reported the first case of Swyer-James-MacLeod syndrome complicated with severe pulmonary hypertension which was diagnosed by right heart catheterization and treated with specific therapy [18].

Treatment is usually conservative including managing and preventing recurrent infections by administering influenza and pneumococcal vaccines [11]. Surgical resection may be essential if infections in the setting of bronchiectasis in affected lung segments cannot be improved with antibiotics and is indicated in case of uncontrollable hemoptysis [8,19]. Prognosis is generally good. However, the coexistence with bronchiectasis has a negative impact on the clinical course of the disease, with patients with bronchiectasis having more severe recurrent infections than those who do not have [19].

## Conclusions

This is one of a handful of cases describing diagnosis of Swyer-James-MacLeod syndrome in adulthood. The syndrome should always be suspected in adults with unilateral hyperlucency or pulmonary emphysema with an atypical distribution on chest X-ray and a history of recurrent lung infections. Investigation of these cases with chest CT may prevent misdiagnosis or a delay in diagnosis, especially in patients who do not respond to conventional therapy, in order to achieve better prognosis and to prevent inappropriate management.
